# Assessing the confidence, knowledge and learning preferences of healthcare workers regarding personal protective equipment use during the coronavirus disease 2019 (COVID-19) pandemic

**DOI:** 10.1017/ice.2022.1

**Published:** 2022-01-06

**Authors:** Rachel Brown, Amanda M. Brown, Sharon Markman, Rukhshan Mian, Vineet M. Arora, Craig A. Umscheid

**Affiliations:** 1 Quality Performance Improvement, University of Chicago Medicine, Chicago, Illinois; 2 Infection Prevention and Control Program, University of Chicago Medicine, Chicago, Illinois; 3 Center for Healthcare Delivery Science and Innovation (HDSI), University of Chicago Medicine, Chicago, Illinois; 4 Center for Health and the Social Sciences (CHeSS), Biological Sciences Division, University of Chicago Chicago, Illinois; 5 Biological Sciences Division, Department of Medicine, University of Chicago, Chicago, Illinois

## Abstract

We surveyed healthcare workers at an urban academic hospital in the United States about their confidence in and knowledge of appropriate personal protective equipment use during the coronavirus disease 2019 (COVID-19) pandemic. Among 461 respondents, most were confident and knowledgeable about use. Prescribers or nurses and those extremely confident about use were also the most knowledgeable.

Appropriate use of personal protective equipment (PPE) by healthcare workers (HCWs) is critical to preventing transmission of severe acute respiratory syndrome coronavirus 2 (SARS-CoV-2).^1–3^ Although studies have examined HCW knowledge of appropriate PPE practices in the context of the coronavirus disease 2019 (COVID-19) pandemic outside the United States, to our knowledge no evaluation of HCWs in the United States has been published.^4,5^ In this study, we examined the confidence, knowledge and learning preferences of HCWs with regard to PPE practices during the COVID-19 pandemic in an urban academic hospital in the United States.

## Methods

A 22-item survey (provided in the Appendix online) was distributed between July 14 and July 31, 2020, to emergency department and inpatient medical center HCWs at the University of Chicago Medical Center, which includes an adult and free-standing children’s hospital with 811 beds and >35,000 inpatient admissions and 100,000 emergency department visits annually. Surveyed HCWs included physician and nurse providers, social workers and care coordinators, nutritionists, chaplains, pharmacists, phlebotomists, therapists, patient transporters, and dialysis and environmental cleaning technicians. The survey was e-mailed to HCWs by their local leadership, and the responses were used to assess demographics, confidence in and knowledge of PPE best practices, and preferences for learning about PPE practices. Responses were captured anonymously using Research Electronic Data Capture (REDCap).^6^ Those who had not worked in the emergency department or on an inpatient hospital unit during the pandemic (between March 15, 2020, and the survey distribution dates) were excluded.

Responses were reported using absolute counts and proportions. Logistic regression examined the association between staff role (ie, prescriber [physician, advanced practice provider], nurse, other), being “high risk” for severe disease (ie, yes, no, prefer not to answer), and confidence in PPE practices (ie, extremely, somewhat, other) with answering all 4 core-knowledge–based questions correctly. “High risk” was defined as those high risk for severe illness from COVID-19, or living with someone who is “high risk,” and included older adults, those with chronic lung disease or moderate-to-severe asthma, diabetes, severe obesity, serious heart conditions, an immunocompromised state, chronic kidney disease, and/or chronic liver disease.^7–9^ Knowledge-based questions were informed by published guidance from relevant organizations locally and globally,^7–9^ and they assessed the following: donning practices prior to entering a room of a patient with COVID-19 or persons under investigation (PUI) for COVID-19 infection (including the need to perform hand hygiene prior to donning, and use of the appropriate mask/respirator, gowns, gloves and eye protection); doffing practices (including doffing gown and gloves prior to leaving a COVID-19 or PUI patient room); appropriate extended use of one’s N95 respirator; and use of an appropriate respirator prior to entering a COVID-19 or PUI room during an aerosol-generating procedures. Analyses were conducted using Stata software (College Station, TX). This project was deemed a formal quality improvement study, and as such it was not reviewed by the institutional review board, which is consistent with institutional policy.

## Results

Of the 531 respondents, 461 (87%) were eligible for inclusion. Most eligible respondents were female; most reported their race as white; >80% worked in adult settings; and up to 30% worked in pediatric settings (Table 1). Most provided direct care to patients with COVID-19 or PUIs, but <20% of respondents provided direct care to patients in the COVID-19 cohort units that were established during the peak of the pandemic, from March 20 to June 24, 2020. Also, >25% of respondents considered themselves or those they lived with “high risk” for severe illness from COVID-19 infection, but few reported being diagnosed with COVID-19. Most were extremely or somewhat confident about PPE use. They correctly answered questions examining core knowledge of PPE best practices; they always or often used isolation signage to facilitate PPE use; and they found PPE isolation signage helpful in facilitating appropriate PPE practices (Table 2).

**Table 1.** Descriptive Characteristics of Survey Respondents

Item and Response	No. (%)
**Role**	
Prescriber (attending, APP, fellow, resident)	160 (34.7)
Nurse	191 (41.4)
Other	110 (23.9)
**Sex**	
Female	327 (70.9)
Male	104 (22.6)
Prefer not to answer	26 (5.6)
**Race**	
White	259 (56.2)
Asian	44 (9.5)
Black	41 (8.9)
More than one race selected	32 (6.9)
Prefer not to answer	85 (18.4)
**Age**	
18–34 y	158 (34.3)
35–44 y	145 (31.5)
≥45 y	143 (31.0)
Prefer not to answer	15 (3.2)
**Provides care to**	
COVID-19 cohort unit patients	77 (16.7)
No COVID-19 or PUI patients	51 (11.1)
COVID-19 or PUI patients, but no cohort unit patients	333 (72.2)
**Work on following units**	
Adult medical surgical	215 (82.4)
Adult intensive care	173 (66.3)
Adult emergency department	90 (34.5)
Pediatric medical surgical	63 (24.1)
Pediatric intensive care	78 (29.9)
Pediatric emergency department	61 (23.4)
Family birth center	57 (21.8)
Operating rooms	56 (21.5)
**High risk for severe COVID-19**	
No	305 (66.2)
Yes	127 (27.5)
Prefer not to answer/Blank	29 (6.3)
**Diagnosed with COVID-19**	
No	431 (93.5)
Yes	8 (1.7)
Prefer not to answer/Other	22 (4.8)

Note. APP, advanced practice provider; COVID-19, coronavirus disease; PUI, person under investigation. “High risk” denotes those who are at high risk for severe illness from COVID-19, or live with someone who is at high risk, and includes older adults, those with chronic lung disease or moderate to severe asthma, diabetes, severe obesity, serious heart conditions, an immunocompromised state, chronic kidney disease, and/or chronic liver disease.

**Table 2.** Survey Items Assessing Confidence, Knowledge and Learning Preferences

Item and Response	No. (%)
**Confident about PPE use**	
Extremely	180 (39.0)
Somewhat	209 (45.3)
Neutral	28 (6.1)
Not confident	37 (8.0)
Extremely not confident	7 (1.5)
**Proper steps for donning PPE prior to room entry**	
Correct	355 (77.0)
Incorrect	81 (17.6)
Do not know	25 (5.4)
**Where to doff when leaving room**	
Correct	389 (84.4)
Incorrect	55 (11.9)
Do not know	17 (3.7)
**If I remove my N95 respirator, I can reuse**	
Correct	325 (70.5)
Incorrect	84 (18.2)
Do not know	52 (11.3)
**Selecting respirator to safely enter room of COVID-19 PUI undergoing AGP**	
Correct	445 (96.5)
Incorrect	6 (1.3)
Do not know	10 (2.2)
**Use signage to facilitate correct use of PPE**	
Always	173 (37.5)
Often	98 (21.3)
Initially, not currently	98 (21.3)
Only when COVID-19 isolation precautions present	85 (18.4)
Only when in COVID-19 cohort unit	7 (1.5)
**Following is most helpful to understand COVID-19–related PPE practices**	
Signage	353 (76.6)
E-mail	214 (46.4)
Huddles	130 (28.2)
Observers	95 (20.6)
Videos	93 (20.2)
Town halls	57 (12.4)
Other	36 (7.8)

Note. AGP, aerosol-generating procedure; COVID-19, coronavirus disease; PPE, personal protective equipment; PUI, person under investigation.

Being extremely confident about PPE use was significantly associated with answering all 4 knowledge-based questions correctly (odds ratio [OR], 1.85; confidence interval [CI], 1.37–2.50; *P* < .001), as was having a role as a prescriber or nurse (OR, 1.64; CI, 1.02–2.63; *P* = .04). Being or living with someone at “high risk” for severe COVID-19 was not significantly associated with answering all knowledge-based questions correctly (OR, 0.85; CI, 0.60–1.19; *P* = .34).

## Discussion

In a convenience sample of emergency department and inpatient HCWs in an urban academic hospital in the United States, most were confident and knowledgeable about PPE use in the context of COVID-19, and they found PPE signage helpful in facilitating appropriate PPE use. Those who were prescribers or nurses, and those who were extremely confident about PPE use were most knowledgeable about PPE practices.

Confidence about and core knowledge of appropriate PPE practices appeared to be greater in our study than in studies of HCWs outside the United States. For example, ∼50% of respondents in a study of HCWs in an academic pediatric hospital in Canada correctly identified the appropriate sequence of donning PPE compared to 77% of respondents in our evaluation,^4^ despite assessing knowledge at similar time points in the global pandemic (ie, mid-2020) and including a similar distribution of prescribers and nurses and respondents providing care in the emergency and intensive care settings. Confidence and core knowledge were also higher in our study compared to estimates reported in a nationwide survey of nurses in the United Kingdom.^5^ The higher estimates reported in our study could be due to remaining differences between our respondents and those in the other studies referenced, such as the inclusion in our study of more providers specializing in the care of adults and exclusively providing care in an academic setting. Differences in approaches to training may also have resulted in greater confidence and knowledge. For example, our institution used a multimodal approach to educating HCWs about PPE practices, including e-mail communications, instructional videos, town halls, team huddles, in-person observers, and isolation precaution signage developed using principles of human-centered design.^10^

Our study had several limitations. Our estimates were from a single site, and responses were self-reported. In addition, our statistical analyses were unadjusted. However, our study did include a larger sample size than those of other similar evaluations,^5,6^ and we included a diverse set of respondents. Thus, our findings may be generalizable to other urban academic medical centers in the United States.

Further studies should examine PPE knowledge among more representative samples of clinical and nonclinical HCWs in the United States, including enriched samples of nonclinical HCWs who have frequent contact with patients, such as food services staff. Future studies should also examine associations between PPE knowledge and appropriate use of PPE.

## Supplementary material

For supplementary material accompanying this paper visit https://doi.org/10.1017/ice.2022.1. click here to view supplementary material
