# Supplementary material for: Assessing the confidence, knowledge and learning preferences of healthcare workers regarding personal protective equipment use during the coronavirus disease 2019 (COVID-19) pandemic
Source: Infect Control Hosp Epidemiol. 2022 Jan 6:1–4. doi: 10.1017/ice.2022.1 (PMC8888000; doi:10.1017/ice.2022.1)
Supplement: Supplementary file 1 [file S0899823X22000010sup001.pdf]

UChicago Medicine's Covid-19 PPE Practices Survey

Tell us what you think!

By completing this survey, you will help UChicago Medicine understand the impact of the resources provided to help faculty and staff follow the PPE practices put in place in response to Covid-19. The survey should take no longer than 10 minutes to complete. Thank you for your time!

---

1. I have been on patient units in the hospital (CCD, Mitchell, ED, Comer) at least once since March 15th, 2020:

◯ No
◯ Yes

---

2. Role (select from drop down list):

◯ APP (NP, PA)
◯ Attending (MD, DO)
◯ Care Coordination
◯ Chaplain/Spiritual Care Department
◯ Clinical Nutrition
◯ Cohort Unit Observer
◯ Cohort Unit Runner
◯ Environmental Services
◯ Fellow
◯ Food Services
◯ Leadership (Manager, Director, etc.)
◯ Medical Student
◯ Nurse
◯ Patient Transport
◯ Pharmacist
◯ Phlebotomist
◯ Resident
◯ Respiratory Therapist
◯ Supply Chain
◯ Technician (Pharmacy, Dialysis)
◯ Therapist (Physical, Occupational)
◯ Other

Other (please describe): ______________________________

---

3. I have provided care or services to (select all that apply):

☐ COVID Positive Patients
☐ COVID Patients Under Investigation (PUIs)
☐ COVID Positive Patients on Cohort Unit
☐ All of the Above
☐ None of the Above

---

4. I have worked on the following units (select all that apply):

☐ CCD Med/Surg
☐ CCD ICU
☐ CCD OR
☐ ED
☐ Comer ED
☐ Comer Med/Surg
☐ Comer ICU
☐ Family Birth Center
☐ Mitchell Med/Surg
☐ Other

Other (please describe): ______________________________

5. I am ____________ in knowing what PPE to use in different situations (select response that most closely reflects your confidence level):

   Example situations include: entering a patient's room with Special Respiratory Precautions and Contact Precaution signs, entering the cohort unit, etc.

   ◯ Extremely confident
   ◯ Somewhat confident
   ◯ Neutral ◯ Not confident
   ◯ Extremely not confident

6. Please select the proper steps for DONNING PPE prior to entering a COVID PUI patient room with Special Respiratory Precautions and Contact Precautions:

   ◯ Perform hand hygiene, DON gloves, DON gown, DON surgical mask/N95/half face respirator/PAPR
   ◯ Perform hand hygiene, DON surgical mask/N95/half face respirator/PAPR, DON gown, DON eye protection, and DON gloves
   ◯ Perform hand hygiene, DON eye protection, DON gloves, DON surgical mask/N95/half face respirator/PAPR
   ◯ DON surgical mask/N95/half face respirator/PAPR, DON gown, DON eye protection, and DON gloves
   ◯ I do not know the answer

7. A patient is on Special Respiratory Precautions and Contact Precautions. When leaving this patient's room, where should you DOFF your gown and gloves? (select the correct location):

   ◯ Outside the patient room
   ◯ Inside the patient room
   ◯ Either inside or outside the patient room
   ◯ None of the above ◯ I do not know the answer

8. True or False:
   If you remove your N95 from over your nose and mouth, you can re-use this N95.

   ◯ True ◯ False ◯ I do not know the answer
   (Please do not guess answer)

9. You are about to enter a COVID PUI room when you notice a sign on the door that states the patient is currently receiving an aerosol generating procedure.

   You can safely enter the patient room during the procedure while wearing what type of mask/respirator? (select the correct response):

   ◯ N95/half face respirator/PAPR
   ◯ Surgical mask ◯ Cloth mask
   ◯ All of the above ◯ I do not know the answer
   (Please do not guess answer)

10. A N95/Half Face Respirator/PAPR is needed for which of the following aerosol generating procedures (select all that apply):

    ☐ Intubation/extubation
    ☐ CPR ☐ Bronchoscopy
    ☐ Nebulizer treatment
    ☐ BiPAP, CPAP ☐ None of the above
    ☐ All of the above ☐ I do not know the answer
    (Please do not guess answer)

11. I use instructional signage to help me follow the COVID donning and doffing PPE practices correctly (select the answer that most closely applies):

    ◯ Initially, but not currently
    ◯ Only when I work with patients with Special Respiratory Precautions and Contact Precautions ◯ Only when I work on the Cohort Unit ◯ Often ◯ Always

12. Please review the following signage:

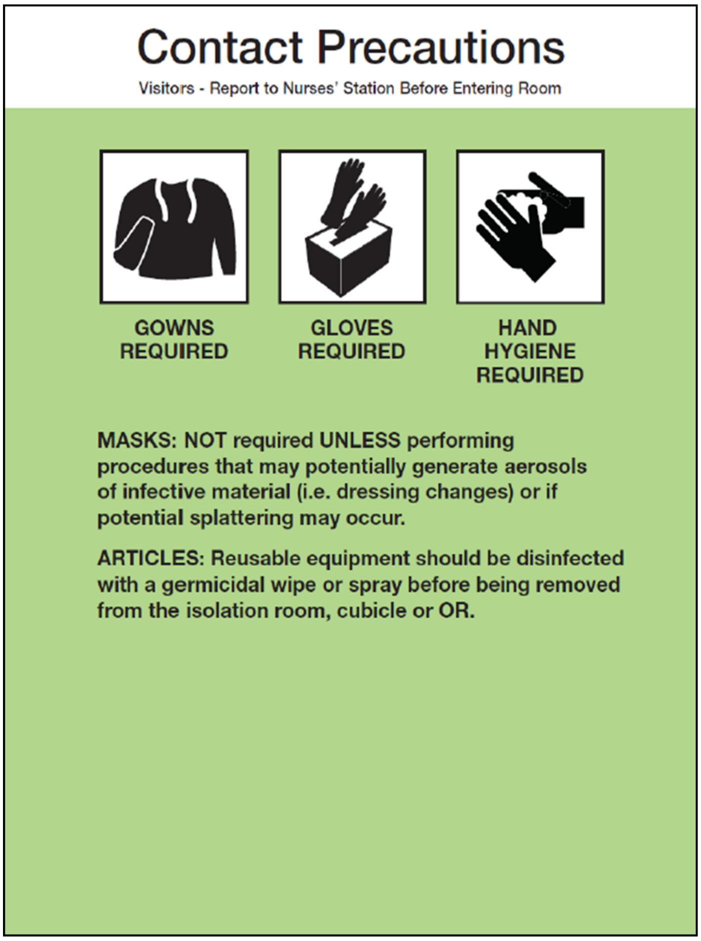

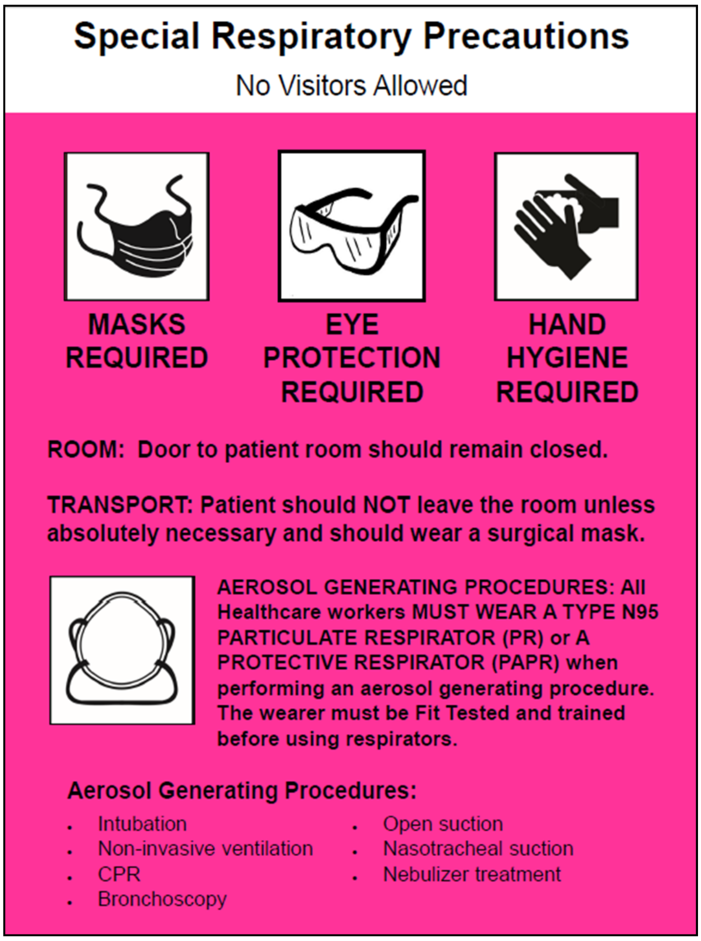

I have seen these signs on patient units:	◯ Yes<br>◯ No
These signs look trustworthy:	◯ Strongly Disagree ◯ Disagree<br>◯ Neutral ◯ Agree ◯ Strongly Agree
These signs are easy to follow:	◯ Strongly Disagree ◯ Disagree<br>◯ Neutral ◯ Agree ◯ Strongly Agree
These signs have informative content on PPE practices:	◯ Strongly Disagree ◯ Disagree<br>◯ Neutral ◯ Agree ◯ Strongly Agree
These signs feature clear imagery and layout:	◯ Strongly Disagree ◯ Disagree<br>◯ Neutral ◯ Agree ◯ Strongly Agree

13. Please review the following signage:

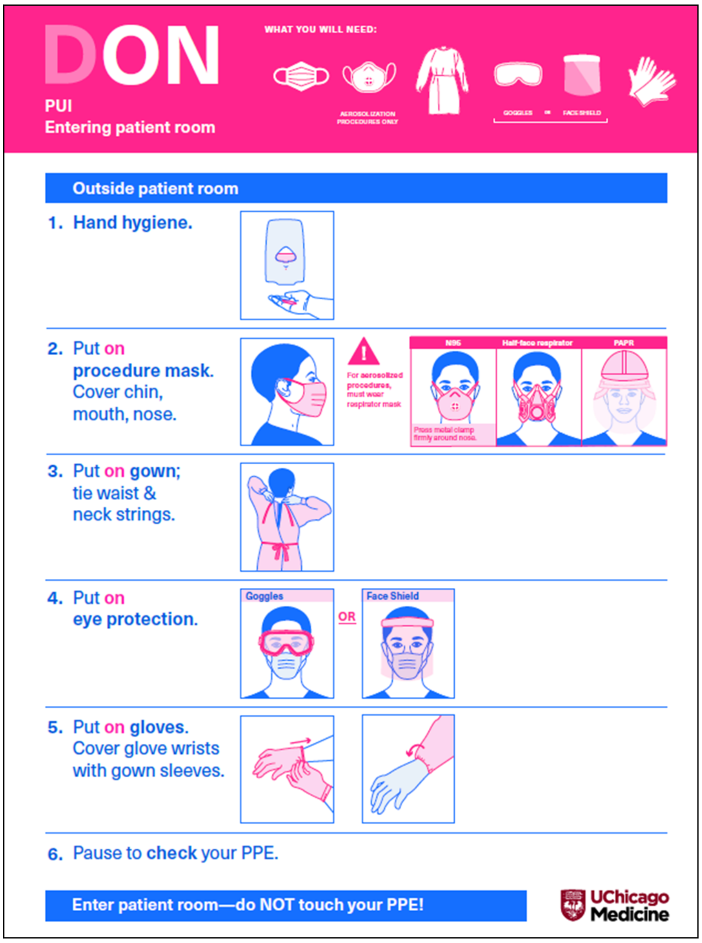

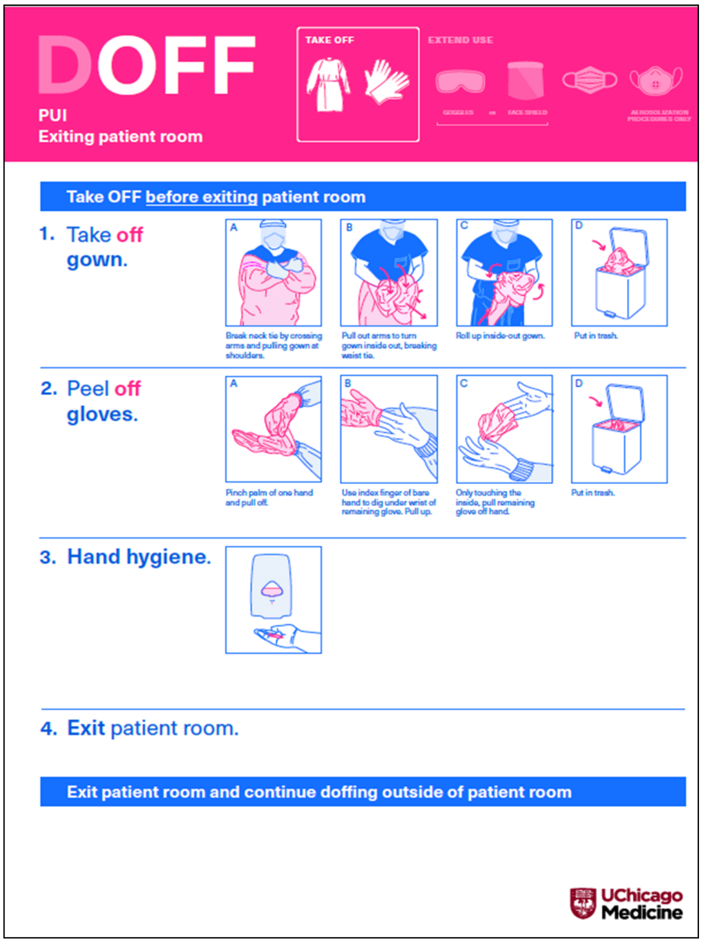

I have seen these signs on patient units:	◯ Yes ◯ No
These signs look trustworthy:	◯ Strongly Disagree ◯ Disagree ◯ Neutral ◯ Agree ◯ Strongly Agree
These signs are easy to follow:	◯ Strongly Disagree ◯ Disagree ◯ Neutral ◯ Agree ◯ Strongly Agree
These signs have informative content on PPE practices:	◯ Strongly Disagree ◯ Disagree ◯ Neutral ◯ Agree ◯ Strongly Agree
These signs feature clear imagery and layout:	◯ Strongly Disagree ◯ Disagree ◯ Neutral ◯ Agree ◯ Strongly Agree

14. Please review the following signage:

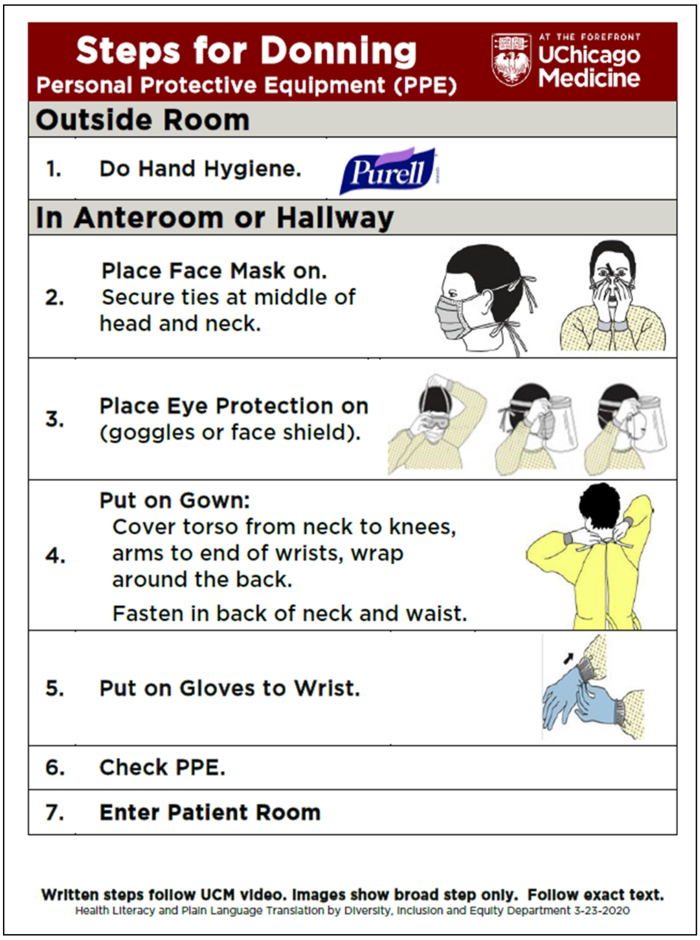

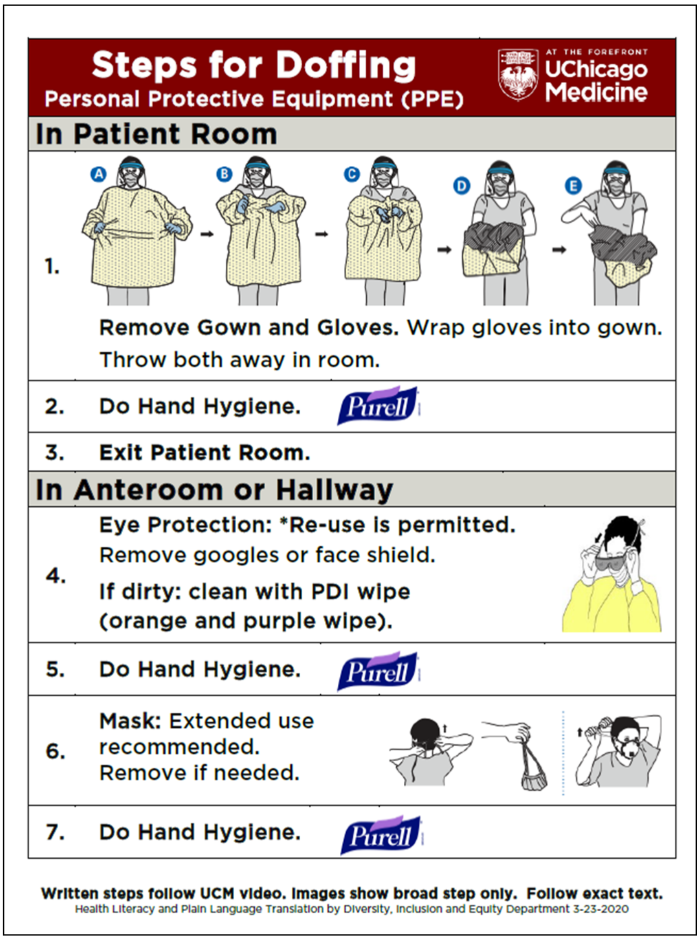

I have seen these signs on patient units:	◯ Yes ◯ No
These signs look trustworthy:	◯ Strongly Disagree<br>◯ Disagree<br>◯ Neutral<br>◯ Agree<br>◯ Strongly Agree
These signs are easy to follow:	◯ Strongly Disagree<br>◯ Disagree<br>◯ Neutral<br>◯ Agree<br>◯ Strongly Agree
These signs have informative content on PPE practices:	◯ Strongly Disagree<br>◯ Disagree<br>◯ Neutral<br>◯ Agree<br>◯ Strongly Agree
These signs feature clear imagery and layout:	◯ Strongly Disagree ◯ Disagree<br>◯ Neutral ◯ Agree ◯ Strongly Agree

15. I have seen these signs on patient units:

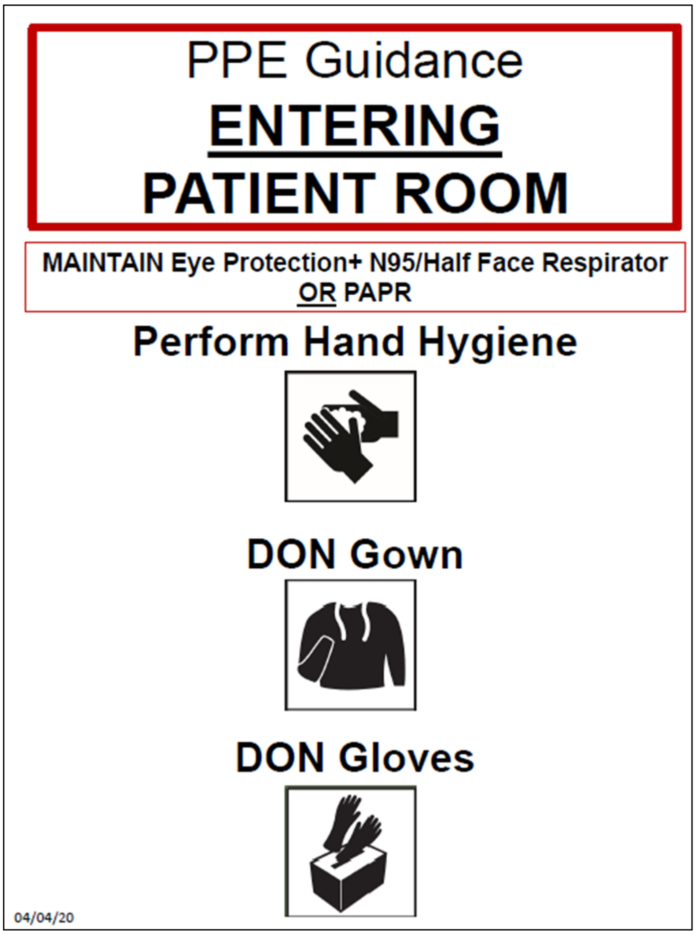

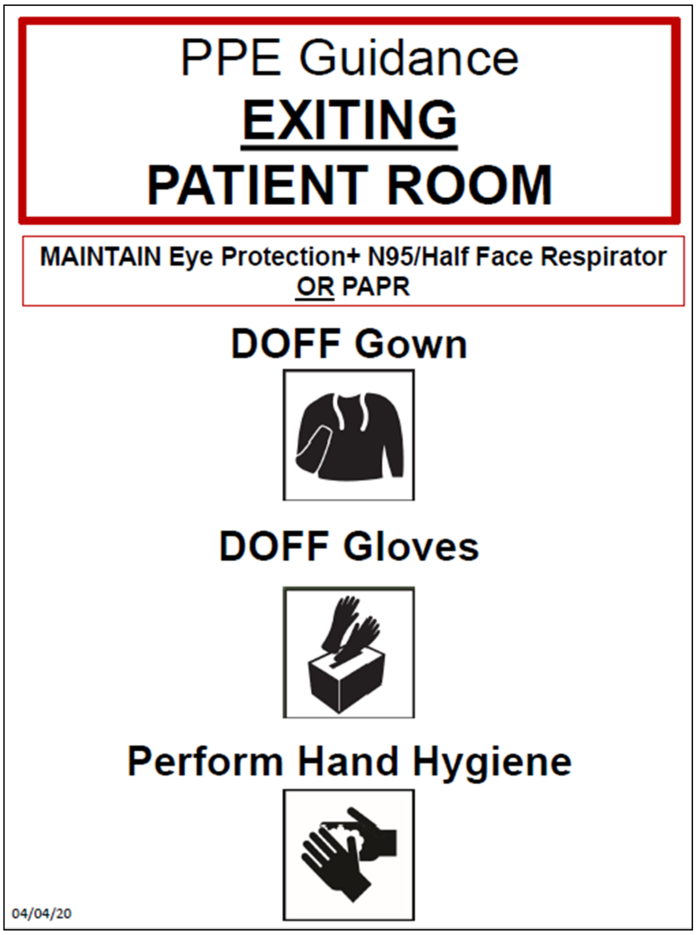

I have seen these signs:	◯ Yes ◯ No
These signs look trustworthy:	◯ Strongly Disagree ◯ Disagree<br>◯ Neutral ◯ Agree ◯ Strongly Agree
These signs are easy to follow:	◯ Strongly Disagree ◯ Disagree<br>◯ Neutral ◯ Agree ◯ Strongly Agree
These signs have informative content on PPE practices:	◯ Strongly Disagree ◯ Disagree<br>◯ Neutral ◯ Agree ◯ Strongly Agree
These signs feature clear imagery and layout:	◯ Strongly Disagree ◯ Disagree<br>◯ Neutral ◯ Agree ◯ Strongly Agree
16. The following have been the most helpful to me in understanding the PPE practices for PUIs and COVID Positive Patients (select all that apply):	☐ Huddles and/or team-level meetings<br>☐ Email communications<br>☐ Visible signage at key areas<br>☐ Videos ☐ Town halls<br>☐ Monitors and/or observers<br>☐ Other
Other (please describe):	__________________________________

17. Please provide feedback on the PPE practice guidance (including written policies, signage) put in place by the hospital in response to the COVID pandemic.

____

18. What is your age category?

◯ 18-24 years old ◯ 25-34 years old
◯ 35-44 years old ◯ 45-54 years old
◯ 55-64 years old ◯ 65+ years old
◯ I prefer not to answer

19. What is your gender?

☐ Male ☐ Female ☐ Other
☐ I prefer not to answer

20. Please specify your race and ethnicity (check all that apply):

☐ American Indian or Alaska Native
☐ Asian ☐ Black or African American
☐ Native Hawaiian or Other Pacific Islander
☐ White ☐ Other ☐ Hispanic or Latino or Spanish Origin ☐ Not Hispanic or Latino or Spanish Origin ☐ I prefer not to answer

21. I am considered high risk for severe illness from COVID or I live with someone who is considered high risk for severe illness from COVID:

NOTE: "High Risk" includes older adults, chronic lung disease or moderate to severe asthma, diabetes, severe obesity, serious heart conditions, being immunocompromised, chronic kidney disease, and/or liver disease.

◯ Yes ◯ No ◯ I prefer not to answer

22. I have been diagnosed with COVID:

◯ Yes ◯ No ◯ I prefer not to answer
◯ Additional Comment(s)

Additional Comment(s):

____
